# Charles Kelman: The Father of Phacoemulsification

**DOI:** 10.7759/cureus.61727

**Published:** 2024-06-05

**Authors:** Abdelaziz A Awad, Hamad A Alkorbi, Hashem Abu Serhan

**Affiliations:** 1 Ophthalmology, Faculty of Medicine, Al-Azhar University, Cairo, EGY; 2 Internal Medicine, Qatar University, Doha, QAT; 3 Ophthalmology, Hamad Medical Corporation, Doha, QAT

**Keywords:** intraocular lens, historical vignette, phacoemulsification, cataract surgery, charles kelman

## Abstract

Charles D. Kelman was a brilliant American ophthalmologist who revolutionized cataract surgery by introducing phacoemulsification to replace extracapsular cataract extraction. He used an ultrasonic probe to emulsify and aspirate the lens through a small incision (3-4 mm). Kelman’s technique met initial resistance at first, but it gained global acceptance after proving its safety and effectiveness in the management of cataractous eyes, and it has been the preferred technique until now. Today, the entire surgery is performed in 5-7 minutes. This technique also helped to reduce hospitalization after the surgical removal of a cataract. Kelman is one of the greatest surgeons of the last century.

## Introduction and background

**Figure 1:**
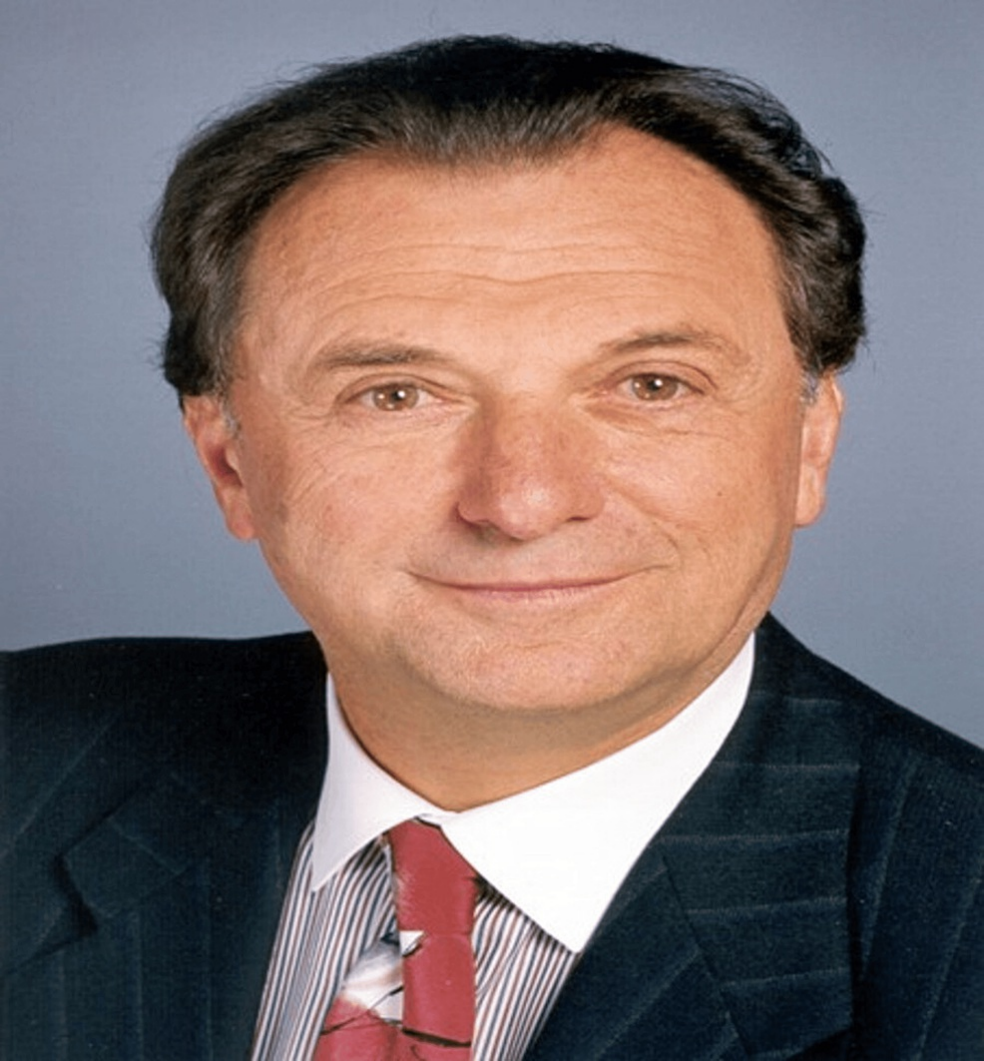
Charles D Kelman, MD (May 23, 1930 – June 1, 2004). Photo taken by Martha Swope. Courtesy of the estate of Charles Kelman. CC BY-SA 4.0

Charles D. Kelman, the father of phacoemulsification, was an American ophthalmologist, born in 1930 in Brooklyn, New York, USA. (Figure 1). He reformed cataract surgery by introducing the phacoemulsification procedure. During this period, he was known for his great efforts in transferring his skills to others and teaching phacoemulsification surgery.

## Review

Kelman’s life and career

Kelman, past president of the American Society of Cataract and Refractive Surgeons, lived from 23 May 1930 to 1 June 2004. He died at the age of 74 due to lung cancer. He was born in New York, USA. He completed his medical studies at Geneva University, Switzerland after graduating from the high school of Forest Hills and the University of Boston’s Tufts. His ophthalmology residency was at the Wills Eye Hospital, in Philadelphia. He worked as a consultant ophthalmologist at many hospitals around the world, in addition to working as an ophthalmology professor at New York Medical College. He worked as an attending surgeon at Manhattan Eye, Ear, and Throat and New York Eye and Ear Infirmary. He loved music and learned to pilot his helicopter [1,2].

Kelman’s innovations

Dr. Kelman developed the cryo-probe technique at the beginning of his career, in which cataractous lenses were frozen, and the capsule was intact after extraction. This technique was the most widely used in the world at that time; however, it was replaced by extracapsular cataract surgery (ECCE) with irrigation and aspiration in 1978 [1]. In ECCE, surgeons remove the entire nucleus of the lens through a large incision (10 mm). Also, he innovated the retinal cryopexy technique to manage patients with retinal detachment in 1963, and this surgery is still applied nowadays [2,3].

Pioneering the phacoemulsification

**Figure 2:**
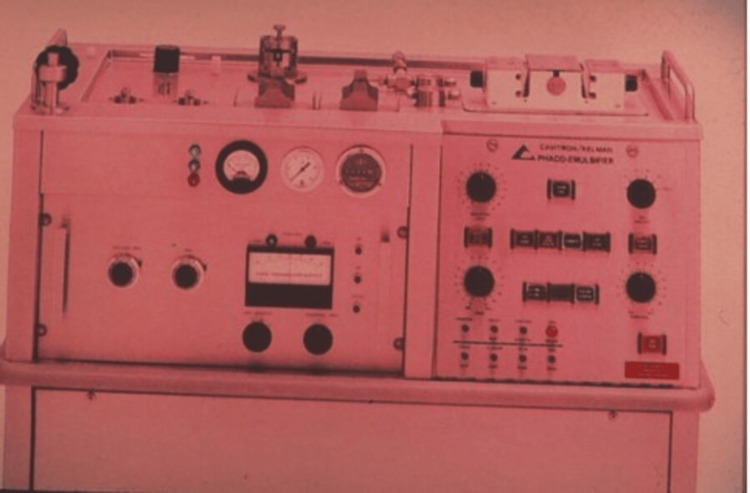
The first production model of the Phacoemulsification unit-c.1971. Photo taken by Norman B. Medow. Photo courtesy C. Kelman. © 2017 Springer International Publishing AG

**Figure 3:**
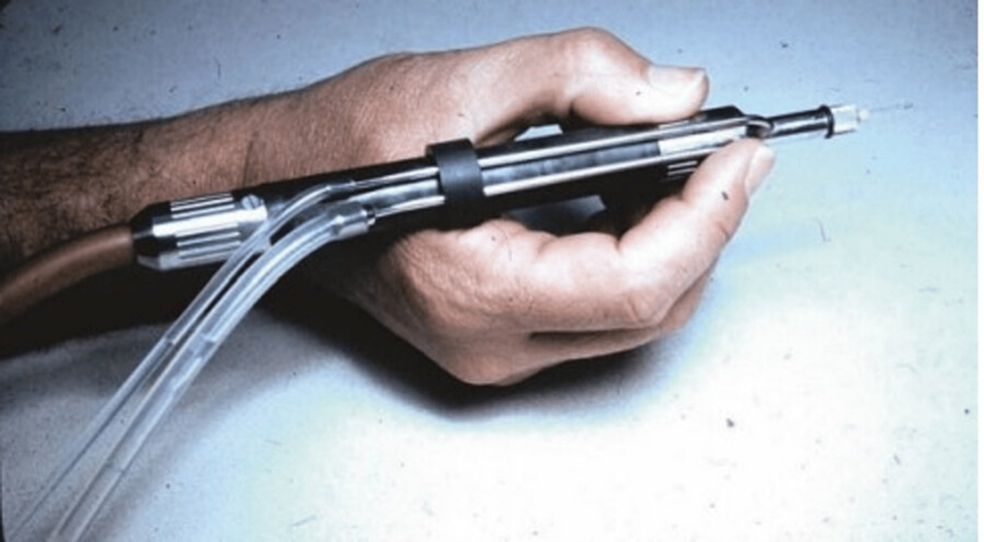
Charlie holding the handpiece of the first phaco production model—c. 1972 Photo taken by Norman B. Medow. Photo courtesy C. Kelman. © 2017 Springer International Publishing AG

Phacoemulsification was first introduced by Dr. Kelman in 1967, and the results were a struggle and initial failure [4-6]. Dr. Kelman wrote a proposal to the Foundation of Hartford to assess outcomes of freezing on the retina, ciliary body, and choroid, also the investigator should apply a small incision to remove the cataract with no need to be hospitalized. He was given a three-year grant, as the head of Hartford Foundation was confident in Kalman’s abilities. Kelman tried everything for two years and eight months to remove the cataract using a folding lens bag, rotating devices, and high-speed cutting needles. All these trials resulted in corneal decompensation. One day, Kelman went to a dentist. He saw an ultrasonic tool that the dentist uses to remove tartar without disturbing the tooth. Asking the dentist what tool he was using; he ran back to his lab to retrieve a recently removed cataract. He took the probe back to the dentist's office and used it to create grooves in the cataract. Kelman thought that he could remove a cataract inside the eye without it vibrating or spinning. An early prototype with a handpiece was made by Cavitron Corporation with aspiration and irrigation (Figures 2, 3). After animal trials, he operated on the first human burned-out glaucoma. During the operation, the cornea collapsed 30-40 times due to the strong surge of suction. Kelman used a device which was manufactured by a company in North Carolina to sense the flow of blood in arteries to prevent corneal collapse after two years. Also, Cavitron Corporation added this feature to the aspiration line of the phacoemulsification device with an air-relief valve. One year later, he operated on a central retinal occlusion patient, complaining of no light perception using a 3D micro-manipulator to stabilize the phaco tip of the eye for extended periods of time and support the weight of the heavy phaco handpiece [6].

The phacoemulsification technique was met with opponents who got the FDA to classify this procedure as experimental. After years of scientific resistance, the head of the American Academy of Ophthalmology (AAO) committee, Richard Troutman, put forward the AAO findings into phacoemulsification, reporting that phacoemulsification had comparable results to the standard of care at that time. During the past three decades, eye surgeons have refined phacoemulsification, and it has become the preferred intervention for eye cataracts. Since 2000, ophthalmologists in the USA have performed phacoemulsification for 97% of surgeries for cataractous lenses [7].

In 1975, Kelman began to design phakic and aphakic lens implants to be used in refractive and cataract eye surgeries, such as Kelman Multiflex and Omnifit [8,9]. He also worked on extracting the cataract using the magnetic technique, which has been used in the removal of arterial plaques and growths from the bladder, prostate, and digestive tract. Phacoemulsification was the motivation trigger for many other microscopic surgeries including neurosurgeries. Kelman's phacoemulsification machine was used by neurosurgeons to remove children's tumors originating from the brain and spinal cord [5].

Awards

Kelman received many awards due to his great contributions to ophthalmology and cataract surgery: the Binkhorst Medal and the First Innovators Award in Ophthalmology (both from the American Society of Cataract and Refractive Surgery), and the Ridley Medal in 1990 by the International Congress of Ophthalmology. He was awarded the first Outstanding Achievement Award for his excellence in cataract surgery from the American Society of Contemporary Ophthalmology. The New York Patent, Trademark and Copyright Law Association awarded him the ‘Inventor of the Year Award’ in 1992 for his development of the Kelman phacoemulsification procedure. President George W Bush awarded him the Prestigious National Medal of Technology. He was named ‘Ophthalmologist of the Century’ at the International Congress on Cataract and Refractive Surgery in Montreal, Canada, due to his great achievements in phacoemulsification. Kelman was honored with the Laureate Recognition award at the 107th Annual Meeting of the AAO in 2003.

## Conclusions

Charles Kelman made a great contribution to ophthalmology, especially cataract surgery through his significant efforts in inventing the phacoemulsification machine, which is considered to be the safest and preferred technique to this day. Kelman’s efforts were not limited to cataract surgery only, but his innovations have contributed to many surgeries on other organs.
